# Myxoid pleomorphic liposarcoma in the teres minor muscle: A case report

**DOI:** 10.1097/MD.0000000000031360

**Published:** 2022-11-04

**Authors:** Jun Ho Choi, Soo Hyuk Lee, Kwang Seog Kim, Yoo Duk Choi, Jae Ha Hwang, Sam Yong Lee

**Affiliations:** a Department of Plastic and Reconstructive Surgery, Chonnam National University Hospital, Chonnam National University Medical School, Gwangju, Korea; b Department of Pathology, Chonnam National University Hospital, Chonnam National University Medical School, Gwangju, Korea.

**Keywords:** liposarcoma, Myxoid, pleomorphic, rotator cuff, teres minor

## Abstract

Myxoid pleomorphic liposarcoma (MPL) is a rare aggressive adipocytic tumor that mainly presents in children and adolescents. It is most frequently observed in the mediastinum and rarely in the head and neck, perineal region, or back. Herein, we report the first published case of MPL of the teres minor muscle.

**Patient concerns:**

A 24-years-old woman presented with a painless palpable mass in her right shoulder.

**Diagnoses:**

Magnetic resonance imaging identified a 9.0 × 7.0 × 4.0 cm mass suspected to be a sarcoma in the teres minor muscle. Positron emission tomography/computed tomography revealed no evidence of distant metastasis. Histopathological examination revealed the mass to be an MPL, which was assigned a histologic grade of 3 according to the French Federation of Cancer Centers Sarcoma Group system. No tumor cells were observed along the resected margins.

**Interventions:**

Under general anesthesia, the right teres minor muscle containing the mass was excised en bloc and frozen biopsy confirmed that the tumor cells did not invade the surrounding tissues.

**Outcomes:**

The patient underwent radiotherapy and was followed up for 6 months without complications.

**Lessons:**

Although MPL in the teres minor muscle is rare, it should be considered in the differential diagnosis in patients with a mass in the teres minor muscle due to its poor prognosis.

## 1. Introduction

First described by Alaggio et al^[1]^ in 2009, myxoid pleomorphic liposarcoma (MPL) is an extremely rare, aggressive adipocytic tumor with a high risk of metastasis and recurrence. MPL has been considered a separate entity in the World Health Organization classification of soft tissue tumors since 2020.^[2]^ MPL has mixed histological characteristics with conventional myxoid and pleomorphic liposarcomas.^[2]^ Genetically, MPL lacks damage-inducible transcript 3 (DIT3) rearrangement and recurrent mouse double minute 2 homolog mouse double minutes 2 homolog (MDM2) amplification.^[2]^

MPL occurs predominantly in female children and adolescents.^[2]^ It is observed most frequently in the mediastinum and rarely in the head and neck, perineal region, or back.^[2]^ However, there have been no reports of MPL in the teres minor muscles. Herein, we report the first published case of MPL of the teres minor muscle.

## 2. Case presentation

**Figure 1.**
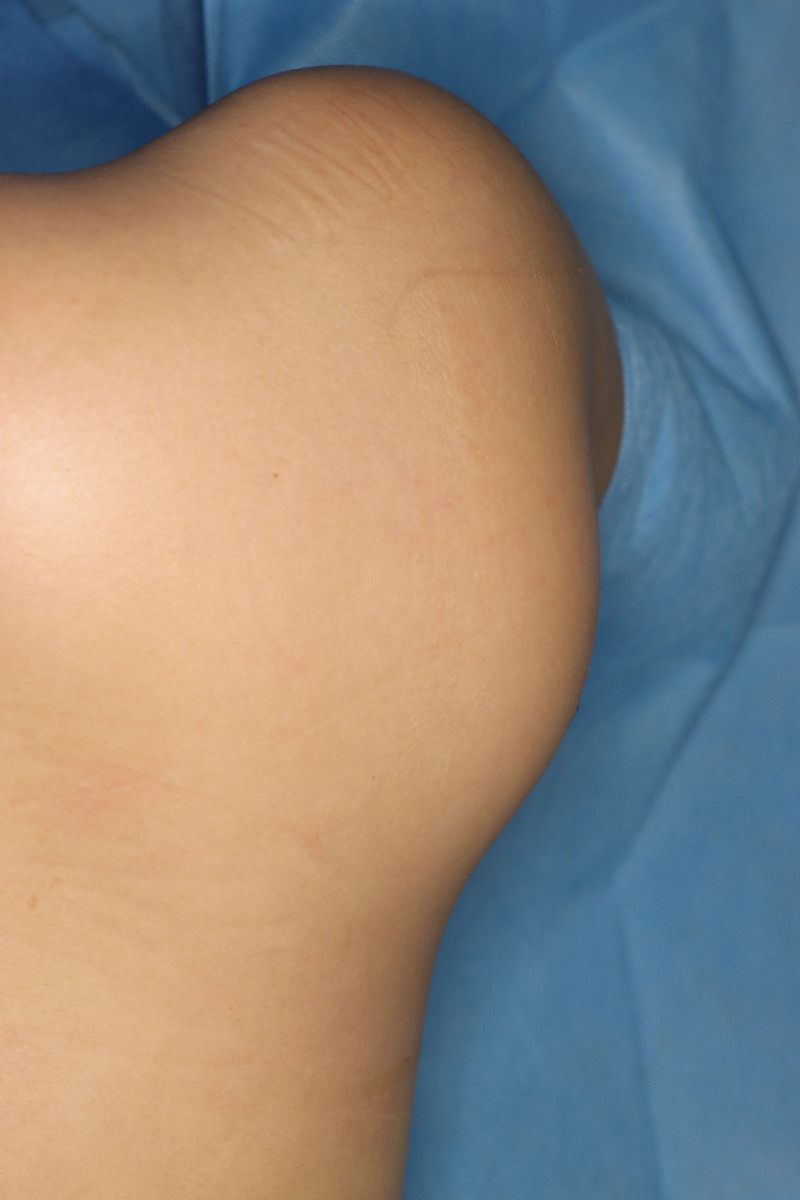
Preoperative photograph of a 24-years-old woman with a mass on her right posterior shoulder.

**Figure 2.**
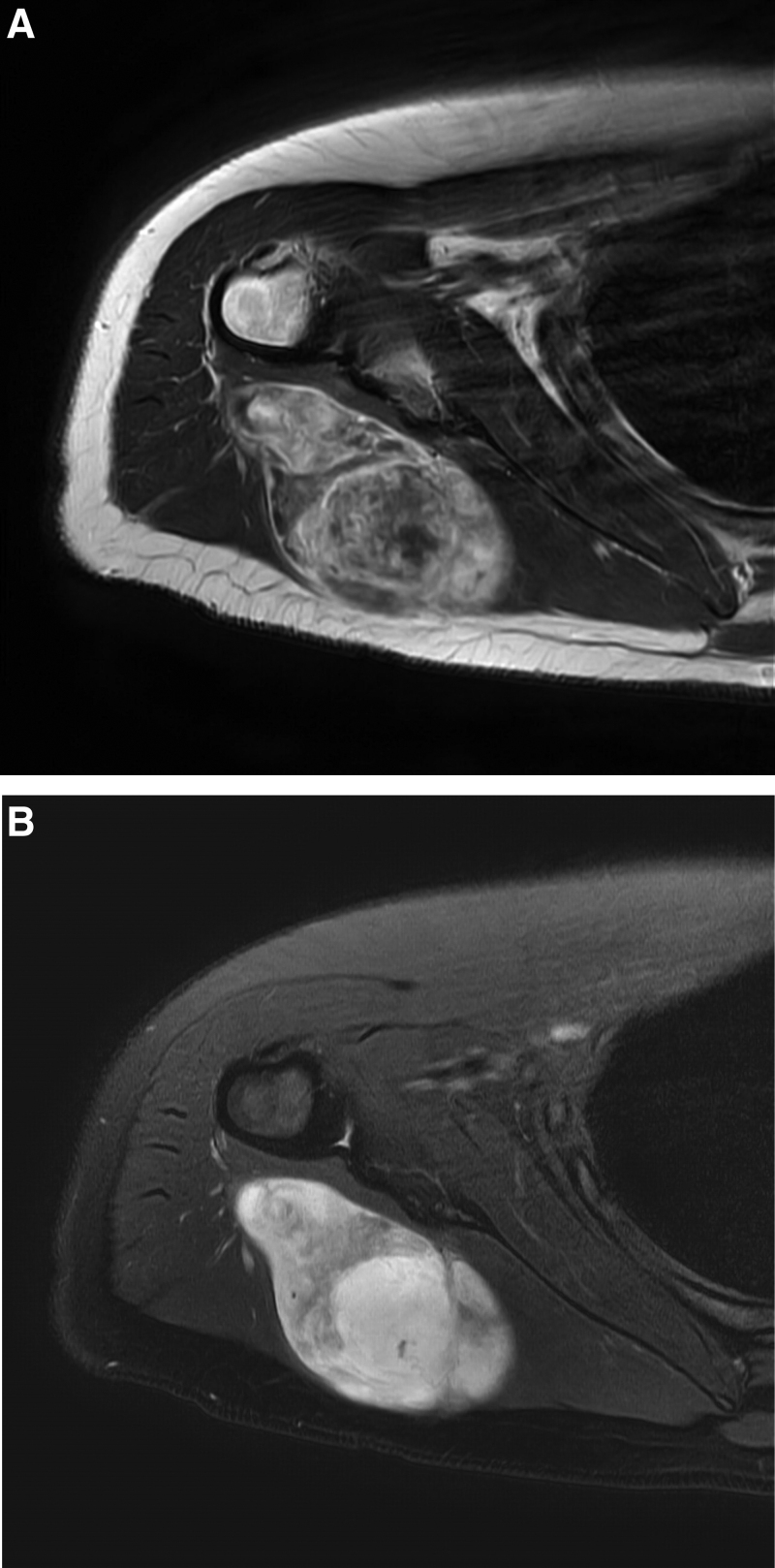
Magnetic resonance imaging showing a 9.0 × 7.0 × 4.0 cm heterogeneous soft tissue mass with a myxoid component in the teres minor muscle. (A) Contrast-enhanced *T1*-weighted imaging. (B) Fat-suppressed *T2*-weighted imaging.

A 24-years-old woman presented with a painless palpable mass in her right shoulder. The mass was detected 4 months prior and grew rapidly (Fig. 1). Shoulder magnetic resonance imaging showed a 9.0 × 7.0 × 4.0 cm mass resembling sarcoma in the right teres minor muscle (Fig. 2). Positron emission tomography/computed tomography showed no evidence of distant metastasis.

The patient had a history of treatment for other tumors twice during childhood. The first tumor was a teratoma in her right ovary at 2 years of age; the second was a rhabdomyosarcoma in her right nostril at 3 years of age. Consequently, the mass in her teres minor muscle was suspected to be a malignant tumor associated with a germline mutation. However, no other family member had a history of cancer, and the test for germline mutations in the TP53 tumor suppressor gene, which are mainly found in Li-Fraumeni syndrome (LFS), was also negative.

**Figure 3.**
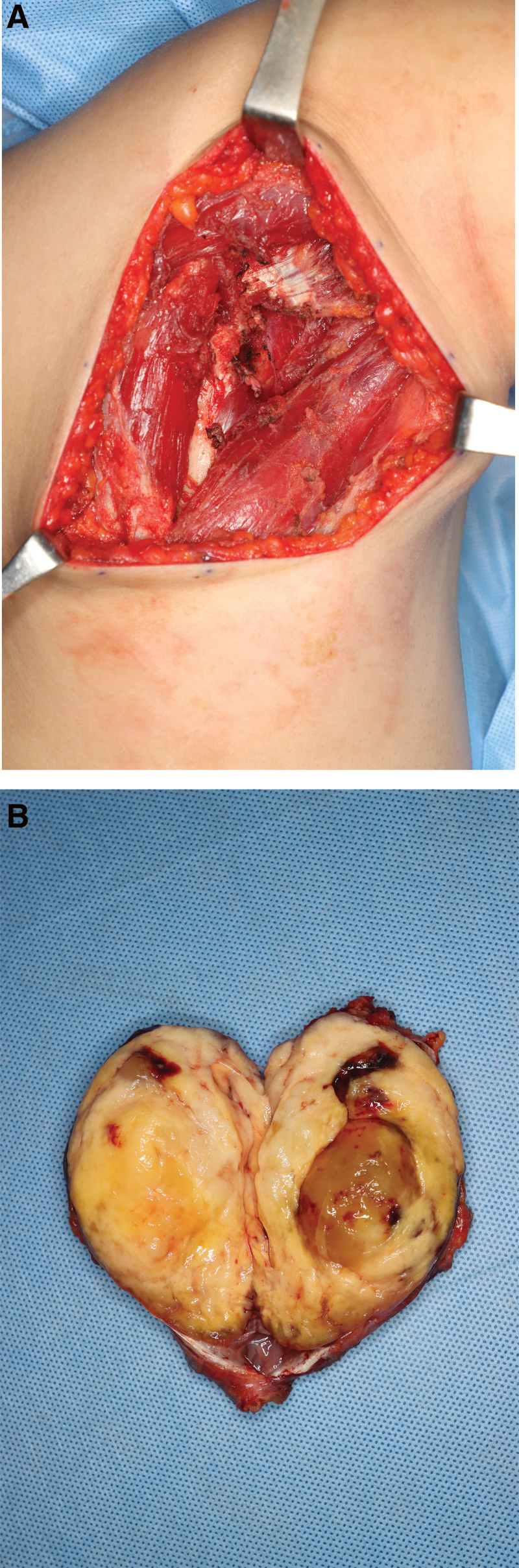
Intraoperative photographs. (A) Operating field after en bloc excision of the mass with the surrounding right teres minor muscle. (B) Cross-section of the mass showing yellow adipose-like appearance in the periphery and myxoid changes in the center.

**Figure 4.**
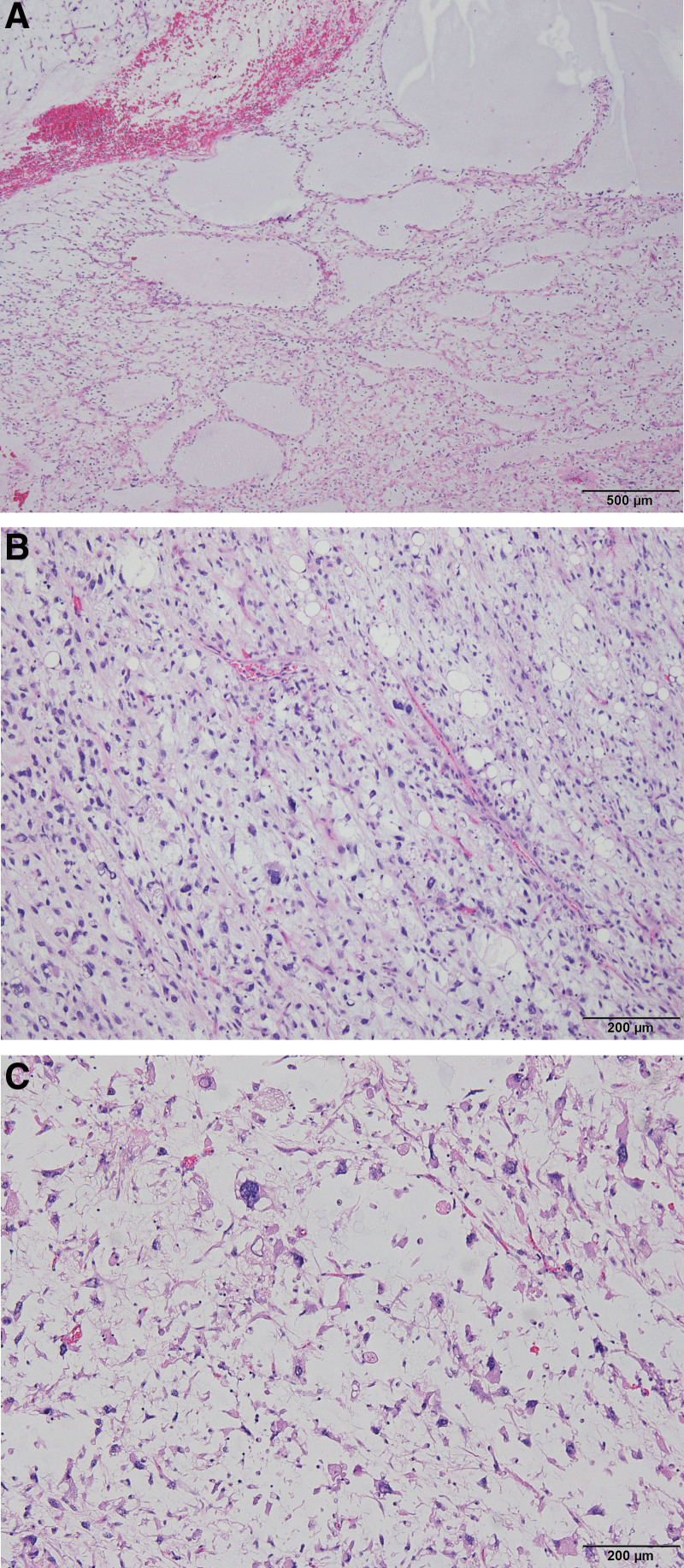
Histopathological examination revealing the mass to be myxoid pleomorphic liposarcoma. Photomicrographs showing (A) lymphangioma-like myxoid pools within the tumor (H&E, ×40); (B) spindle to ovoid primitive tumor cells, lipoblasts, and a delicate curvilinear capillary vasculature (H&E, ×100); and (C) scattered pleomorphic cells and pseudocystic changes (H&E, ×100). H&E = hematoxylin and eosin staining.

Under general anesthesia, the entire area of the right teres minor muscle, including the mass, was excised en bloc, and frozen biopsy confirmed that the tumor cells had not invaded the surrounding tissues (Fig. 3). Histopathological examination revealed the mass to be MPL (Fig. 4). The histologic grade was 3 according to the French Federation of Cancer Centers Sarcoma Group system. No tumor cells were observed along the resected margins. Four cycles of radiotherapy were performed. No recurrence or metastasis was observed for 6 months; however, further workup was not possible as the patient refused evaluation of possible associated malignant tumors and genetic disorders.

## 3. Discussion

Liposarcomas are divided into three histological subtypes: well- and de-differentiated liposarcoma, myxoid/round cell liposarcoma, and pleomorphic liposarcoma.^[3]^ Each subtype is characterized by specific genetic changes that are presumed to induce tumor initiation.^[3]^ MPL is a subtype of liposarcoma recently defined by Alaggio et al^[1]^ in 2009 that shows mixed histological characteristics of conventional myxoid liposarcoma and pleomorphic liposarcoma.^[4]^ MPL shows no fused in sarcoma/Ewing’s sarcoma RNA binding protein 1 (ESWR1)-damage-inducible transcript 3 fusions, as observed in myxoid liposarcoma, and there is no amplification associated with the MDM2 nuclear gene, as observed in well-differentiated or de-differentiated liposarcoma.^[2]^ In the present case, histopathological biopsy showed a mixture of lymphangioma-like myxoid pools and scattered pleomorphic cells with pseudocystic changes (Fig. 4). Our patient showed weak immunoreactivity for S100, Ki-67, and integrase interactor 1. Cluster of differentiation (CD)68, CD34, and MDM2 were unreactive.

Most MPLs are large and typically occur in young women.^[2]^ MPL occurs in the mediastinum, head and neck, extremities, abdominal cavity, and trunk.^[5]^ MPL is associated with high local recurrence, distant metastasis, and low survival rates.^[6]^ Furthermore, age ≥ 60 years, non-extremity lesions, deep tumors, and large tumors (diameter ≥ 5 cm) are associated with poor prognosis.^[6]^ However, there are no agreed-upon recommendations in the management standards associated with MPL.^[5]^ In our case, the MPL measured 9.0 × 7.0 × 4.0 cm and was located on the shoulder of a 24-years-old woman. Similar to the surgical treatment of liposarcoma, the tumor was removed en bloc with the surrounding teres minor muscle, with no tumor cells observed in the resected margin. Although frozen biopsy confirmed that the tumor cells did not invade the surrounding tissues, radiotherapy was performed to prevent local metastasis. No recurrence or metastasis was observed for 6 months.

Soft tissue tumors are overwhelmingly benign, with lipomas predominating.^[7]^ Lipomas are the most common soft tissue tumors and occur in various regions of the body, including the shoulder.^[8,9]^ However, while malignant tumors can occur in the shoulder, such as liposarcoma, myxofibrosarcoma, pleomorphic undifferentiated sarcoma, dermatofibrosarcoma protuberans, synovial sarcoma, leiomyosarcoma, and malignant peripheral nerve sheath tumors,^[7]^ malignant tumors in the teres minor muscles are rare. A literature search of the Ovid, PubMed, Scopus, and Web of Science electronic databases on June 30, 2022, using the terms teres minor, sarcoma, carcinoma, cancer, malignant tumor, and malignancy and without date or language restriction, revealed no other reports.

The rotator cuff is a muscle group composing the supraspinatus, infraspinatus, subscapularis, and teres minor. This muscle group stabilizes the shoulder joint and centers the humeral head in the glenoid cavity. Although the teres minor muscle primarily provides external rotation of the shoulder joint, the infraspinatus muscle is the main external rotator of the shoulder joint. Therefore, if the infraspinatus muscle is healthy, the absence of the teres minor muscle has little effect on shoulder joint function.^[10]^ Following the en bloc resection of the MPL surrounded by the teres minor muscle, the patient did not complain of shoulder function discomfort and did not request further evaluation or treatment.

Several case reports have described an association between MPL and LFS in adolescents and young adults.^[11–13]^ The characteristic tumors in the LFS spectrum include soft tissue sarcomas, osteosarcomas, brain tumors, premenopausal breast cancers, adrenal cortical carcinomas, and leukemias.^[14]^ The patient in the present case had a history of teratoma in the right ovary at 2 years of age, rhabdomyosarcoma in the right nostril at 3 years of age, and MPL in the teres minor muscle at 23 years of age. However, analysis of a blood sample did not show a germline mutation in TP53, and the patient’s family history did not correspond to the LFS criteria. A relationship between complex chromosomal alterations and MPL has also recently been reported.^[4]^ While additional whole-genome sequencing tests were recommended to detect other genetic disorders, the patient refused.

This is the first case report of an MPL in the teres minor muscle. Although MPL in the teres minor muscle is rare, due to its poor prognosis, this condition should be considered in the differential diagnosis in patients with a mass in the teres minor muscle.

## Author contributions

**Conceptualization:** Kim KS.

**Data curation:** Choi JH, Lee SH.

**Formal analysis:** Choi YD, Hwang JH, Lee SY.

**Methodology:** Choi YD, Hwang JH, Lee SY.

**Project administration:** Kim KS.

**Investigation:** Choi JH. Lee SH.

**Writing - original draft:** Choi JH, Lee SH, Kim KS, Choi YD, Hwang JH, Lee SY.

**Writing - review & editing:** Kim KS.
